# Genome Sequencing and Comparative Genomics of Indian Isolates of *Brucella melitensis*

**DOI:** 10.3389/fmicb.2021.698069

**Published:** 2021-08-20

**Authors:** Kumaragurubaran Karthik, Subbaiyan Anbazhagan, Prasad Thomas, Murugesan Ananda Chitra, Tuticorin Maragatham Alagesan Senthilkumar, Ramaswamy Sridhar, Gopal Dhinakar Raj

**Affiliations:** ^1^Central University Laboratory, Tamil Nadu Veterinary and Animal Sciences University, Chennai, India; ^2^Division of Bacteriology and Mycology, Indian Veterinary Research Institute, Bareilly, India; ^3^Translational Research Platform for Veterinary Biologicals, Tamil Nadu Veterinary and Animal Sciences University, Chennai, India; ^4^Centre for Animal Health Studies, Tamil Nadu Veterinary and Animal Sciences University, Chennai, India

**Keywords:** *Brucella melitensis*, India, pangenome, virulence factors, comparative genomics, vaccine candidate

## Abstract

*Brucella melitensis* causes small ruminant brucellosis and a zoonotic pathogen prevalent worldwide. Whole genome phylogeny of all available *B. melitensis* genomes (*n* = 355) revealed that all Indian isolates (*n* = 16) clustered in the East Mediterranean lineage except the ADMAS-GI strain. Pangenome analysis indicated the presence of limited accessory genomes with few clades showing specific gene presence/absence pattern. A total of 43 virulence genes were predicted in all the Indian strains of *B. melitensis* except 2007BM-1 (*ricA* and *wbkA* are absent). Multilocus sequence typing (MLST) analysis indicated all except one Indian strain (ADMAS-GI) falling into sequence type (ST 8). In comparison with MLST, core genome phylogeny indicated two major clusters (>70% bootstrap support values) among Indian strains. Clusters with <70% bootstrap support values represent strains with diverse evolutionary origins present among animal and human hosts. Genetic relatedness among animal (sheep and goats) and human strains with 100% bootstrap values shows its zoonotic transfer potentiality. SNP-based analysis indicated similar clustering to that of core genome phylogeny. Among the Indian strains, the highest number of unique SNPs (112 SNPs) were shared by a node that involved three strains from Tamil Nadu. The node SNPs involved several peptidase genes like U32, M16 inactive domain protein, clp protease family protein, and M23 family protein and mostly represented non-synonymous (NS) substitutions. Vaccination has been followed in several parts of the world to prevent small ruminant brucellosis but not in India. Comparison of Indian strains with vaccine strains showed that M5 is genetically closer to most of the Indian strains than Rev.1 strain. The presence of most of the virulence genes among all Indian strains and conserved core genome compositions suggest the use of any circulating strain/genotypes for the development of a vaccine candidate for small ruminant brucellosis in India.

## Introduction

Brucellosis is an important re-emerging infectious and zoonotic disease worldwide caused by bacterial species within the genus *Brucella* (Martins Rda et al., 2012). Brucellosis goes hand in hand with the agrarian community, who are engaged in the practice of sheep herding (Mantur and Amarnath, 2008). Due to its infectious nature, the pathogen has been recognized as a potential agent for biological warfare (Doganay and Doganay, 2013). Small ruminant brucellosis is considered as an economically important disease of sheep and goat, caused by *Brucella melitensis* (Blasco and Molina-Flores, 2011). Apart from sheep and goat, *B. melitensis* can infect a wide range of mammals like cattle, dog, pig, camel, and wild animals causing abortion and infertility (Office International des Epizooties (OIE), 2018; Simpson et al., 2021). Ever since its identification in the year 1887 by Bruce, enormous research has been carried out on this pathogen, but still, there is no effective vaccine or adequate control measures to eradicate the disease worldwide (Alton, 1990). Due to its zoonotic potential, vaccination among small ruminants is practiced using live attenuated strain Rev.1 in several parts of the world. Adverse effects like abortion in pregnant animals, secretion of the organism in milk and potential to infect humans due to mishandling hamper the use of this vaccine globally (Bardenstein et al., 2002). *B. melitensis* M5 strain derived from *B. melitensis* M28 is used as a vaccine in both non-pregnant and pregnant goat and sheep in China (Jiang et al., 2013). In India, vaccination of small ruminants is not officially recommended, and hence, vaccination is not done against *B. melitensis* in India (Shome et al., 2014).

Epidemiological surveillance tool like multilocus sequence typing (MLST) has been used as a typing tool for various bacteria including *Brucella* spp. MLST is applicable for predicting circulating genotypes and also for outbreak investigations (Khan et al., 2021). However, MLST may not completely discriminate between strains since it does not cover the entire genome of the bacteria (Tsang et al., 2017). Whole genome sequence data represent the complete gene repertoire and, hence, can differentiate strains even from a single outbreak or those in circulation in a particular geographical area (Schaeffer et al., 2021). The core genome alignment-based phylogeny or SNP-based phylogeny deduced from genome sequence data and comparative genomics are applicable for discrimination of strains. Of these, phylogeny based on pangenome SNPs will cover all regions of genome including intergenic regions and, hence, covers more genome regions compared with MLST or core genome phylogeny (Rajendhran, 2021). Molecular surveillance of pathogens is essential to identify the antigenic changes so as to develop a better vaccine candidate. This approach, hence, will aid in the detection of even minor changes in closely related strains, thus, aiding in the development of a vaccine candidate.

In the year 2002, the first genome of *Brucella* sp. was completed, and presently, there are 355 genome sequences of *B. melitensis* available in NCBI. Even though genomic data on this important pathogen are available, molecular pathogenesis is not clearly understood. Hence, control measures cannot be effectively framed. Comparative genomics of the available strains can help to identify the relationship between the *B. melitensis* worldwide, and it can reveal the genes responsible for virulence and pathogenesis of the bacteria. Since vaccination against small ruminant brucellosis is not followed regularly in India, and there is a need for a suitable vaccine candidate, initially, it is essential to know the genomic features, virulence factors, and genetic diversity of *B. melitensis* strains from India so that efforts can be focused on the selection of a better vaccine candidate. Hence, comparative genomics of all available Indian strains (*n* = 17) was carried out in the present study to know their relatedness with vaccine strains (Rev.1 and M5) used worldwide.

## Materials and Methods

### Clinical Presentation, Strain Isolation, and Confirmation

The bacterial strain used in the present study (TN_CUL_1) was isolated from sheep (*Ovis aries*) fetal aborted stomach content in the year 2017 from Perambalur district of Tamil Nadu, India. Abortions were reported in a nomadic sheep population with a flock strength of 400 animals. Most of these abortions in sheep were recorded in the late stage of pregnancy. Around 10 abortions were reported within a period of 2 months, and hence, samples like aborted fetal stomach contents, placenta, and serum from affected animals were collected and processed for diagnosis. All the samples were processed on blood agar, Brucella selective agar, MacConkey agar, and suspected colonies were subcultured on blood agar. Colonies suggestive of *Brucella* were subjected to Gram staining, modified acid-fast staining, and biochemical tests. Suspected colonies were confirmed using AMOS-PCR as per Bricker and Halling (1994).

### Genome Sequencing, Assembly, and Annotation

Genomic DNA of TN_CUL_1 was isolated by Xcelgen bacterial gDNA isolation kit. Quality of DNA was checked on 0.8% agarose gel, and concentration was estimated using Qubit^®^ 2.0 Fluorometer. Genome sequencing was carried out using Illumina© technology at Xcelris Labs Limited, Ahmedabad-380015, Gujarat, India. Briefly, library preparation was carried out using Truseq Nano DNA Library prep kit, and sequencing was done in Illumina platform for the generation of 2 × 150-bp paired end read data. Most of the bioinformatic analysis was carried out in Galaxy (homepage^1^, main public server^2^) (Afgan et al., 2018). The quality of raw reads was assessed by FastQC^3^ tool. Genome assembly was carried out using Unicycler version 0.4.8.0 (Wick et al., 2017). ABACAS version 1.3.1 was used to reorder the contigs based on *B. melitensis* 16 M reference strain into two chromosomes (Assefa et al., 2009). Genome annotation was carried out using Prokka 1.14.5 (Seemann, 2014), NCBI Prokaryotic Genome Annotation Pipeline (Tatusova et al., 2016), and Rapid Annotations using Subsystems Technology (RAST) annotation pipeline (Aziz et al., 2008).

### Data Retrieval and Global Clustering

The available genome assembly data for *B. melitensis* (355) as of January 26, 2021 from NCBI were retrieved (Supplementary Table 1). Details of Indian strains available among global strains detailing the host, geographical, year of isolation, and accession numbers are detailed in Supplementary Table 1. To infer the phylogenetic relatedness of Indian strains among the global strains, a whole genome alignment was made using realphy online webserver^4^. Best model and method were identified and phylogeny was constructed with GTR + G substitution model and maximum likelihood approach (1,000 bootstrap replications) in IQTREE software (Trifinopoulos et al., 2016). Phylogeny was visualized and annotated with iTOL online tool (Letunic and Bork, 2007).

### Genome Features and Pangenome Analysis

Genomic features for virulence factors were predicted using ABRicate (Seemann, 2014) searches of assembled contigs to *Brucella* database in virulence factor database (VFDB) server (Liu et al., 2019) with a minimum 80% DNA identity and coverage. Prophages in the genomes were predicted using Prophage Hunter (Song et al., 2019). The results of the presence or absence of virulence genes and prophages were analyzed and interpreted using Displayr software^5^. The strains used for detailed comparative genomic study targeted all the currently available Indian strains (*n* = 17) and vaccine strains (*n* = 2). Orthologous clustering and pangenome analysis were carried out using Panaroo (Tonkin-Hill et al., 2020). Two-dimensional scaling and visualization of pangenome was carried out using FriPan^6^. The total accessory gene sequences were extracted from pangenome reference.fa file. Further accessory genomes were filtered and scanned for COG annotations in eggnog-mapper tool^7^.

### Multilocus Sequence Typing, Core Genome Phylogeny, and SNP Analysis

Genotypes based on MLST were carried out using pubMLST---brucella based on nine locus schemes^8^ (Whatmore et al., 2007). For inferring the core genome phylogeny, the core alignment file (^∗^.FASTA) based on concatenated core genes from Panaroo was used. The recombination sites predicted by Gubbins were excluded in the phylogeny (Croucher et al., 2015). Pangenome SNP analysis was carried out using kSNP3.0 (Gardner et al., 2015). The optimal size of the nucleotide regions flanking the SNPs (kmer) was identified using Kchooser available with the package. The SNP matrix file (SNPs_all_matrix.fasta) generated by the tool was used to produce a maximum-parsimony tree and for SNP analysis.

## Results and Discussion

Brucellosis caused by *B. melitensis* is one of the major zoonotic disease worldwide; hence, it requires early diagnosis and control. Brucellosis is endemic in India, and a nationwide survey (24,056 small ruminants tested) showed that there was an 11.55% sero-prevalence of brucellosis in sheep, while there was 5.37% sero-prevalence in goats (Shome et al., 2020). The bacterial strain used in the present study (TN_CUL_1) was isolated from a sheep from Tamil Nadu, India. The isolated organism was Gram-negative, catalase, oxidase, and urease positive. The isolate was confirmed to be *B. melitensis* by AMOS PCR. The samples were negative for other infectious agents causing abortion in sheep apart from *B. melitensis*. Genome sequencing followed by assembly of the strain generated 30 contigs representing a genome size of 3,280,927 bp. The GC% of the genome was 57.24%. The schematic representation of chromosome 1 and 2 are shown in Supplementary Figures 1, 2. Prokka and RAST annotated 2,676 and 2,632 protein-coding genes for the strain TN_CUL_1. A total of 51 tRNAs, one 5s rRNA, one 23s rRNA, and one 16s rRNA were predicted in the genome. Similarly, a total of 17 simple sequence repeats (SSRs) or micro-satellites were predicted in the genome of TN_CUL_1. The sequence was submitted in NCBI SRA with the accession number NZ_JACNEQ000000000.1 and assembly number GCA_014270005.1.

**FIGURE 1:**
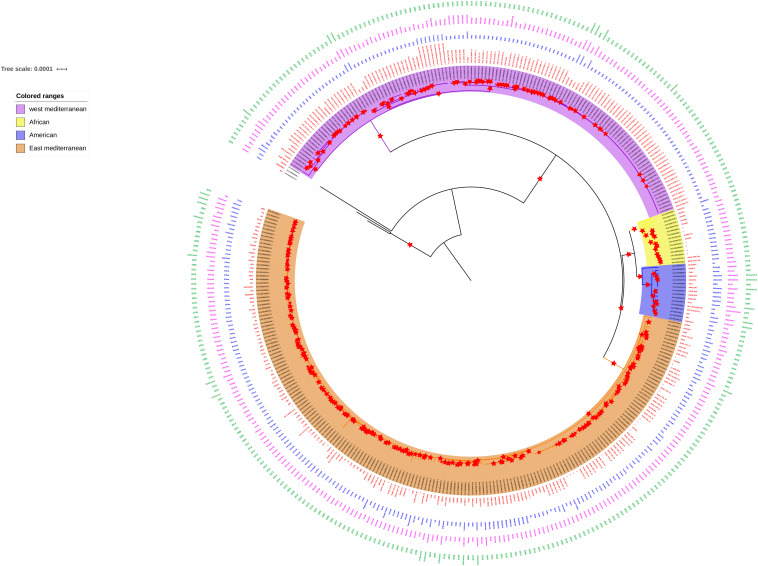
Whole genome phylogeny. Four clusters, namely, West Mediterranean, East Mediterranean, African, and American were grouped in the phylogeny. Most of the Indian strains including TN_CUL_1 fall in the East Mediterranean cluster. Red star symbol indicates boot strap values of more than 80.

Very few studies report the genome analysis and comparative genomics involving Indian *B. melitensis* strains, and only a few strains were compared with the earlier studies (Azam et al., 2016; Kishnani et al., 2018; Singh et al., 2018). The current available whole genome sequence data from India are 17 belonging to different host origins, which also include three strains from sheep (Supplementary Table 1). Recent whole genome study reported the clustering of strains based on geographical origin as Africa, America, and East and West Mediterranean lineages (Janowicz et al., 2020). Since only 10 Indian strains were included in the same study, we created a whole genome phylogeny involving all available Indian strains (*n* = 17) (Supplementary Table 2) along with all available global strains. The whole genome phylogeny indicated that all Indian strains, except ADMAS-G1, clustered together with the East Mediterranean group along with vaccine strain M5 like those in the report of Janowicz et al. (2020). ADMAS-G1 clustered with American lineage along with Rev.1 vaccine strain (Figure 1). Each clade supporting the phylogenetic tree was with sufficient bootstrap values of >80%. The two strains from China, namely, S66 and HN20190002 formed outgroups when compared with other *B. melitensis* isolates worldwide. The possibility of strains S66 and 16M13W not related to *B. melitensis* was reported by earlier workers (Tan et al., 2015; Pisarenko et al., 2018).

### Prophage Diversity and Genetic Variation in Virulence Factors

**FIGURE 2:**
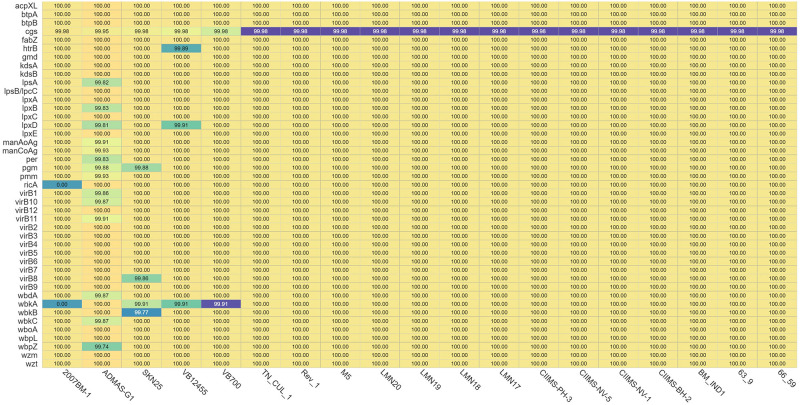
Heatmap depicting the presence and absence of virulence genes. In the 2007BM-1 strain, genes *ricA* and *wbkA* are absent. Numbers in each cell represent the percentage similarity of the nucleotides of each virulence gene with the reference strain 16M.

Prophages play a major role in acquiring novel genes in a bacterium providing new functions to the organism (Wang and Wood, 2016). Virulent/lytic phages have been used for typing *Brucella* spp., but information on temperate phages is very scarce (Hammerl et al., 2016). Hence, *in silico* prediction of prophages was carried for the 19 *B. melitensis* genome to know the diversity of phages present in the genome. TN_CUL_1 is harboring five active/ambiguous prophages, namely, *Moraxella* phage Mcat6 (score 0.71), *Sulfitobacter* phage NYA-2014a, which was predicted at two regions (score 0.88 and 0.93), *Vibrio* phage VpKK5 (score 0.52), and *Brucella* phage BiPBO1 (score 0.91). Except *Vibrio* phage VpKK5 and *Moraxella* phage Mcat6, all other phages were predicted to be active. Among the 19 genomes used in the study, SKN25 strain had a maximum of 22 prophages followed by Rev.1 and LMN18 with 14 prophages (Figure 2). Due to the diversity in prophage content among different strains, it may be employed as an epidemiological tool for tracking the outbreak as reported earlier by Kaden et al. (2014). Prophages in bacteria may have a role in the adaptation of the organism to a wide range of host through transfer of genetic material by the process called transduction (Tormo et al., 2008). Lytic *Brucella* phages have been used for typing *Brucella* spp., but there is limited information of temperate phages of *Brucella*, which has a role in horizontal transfer of genes (Hammerl et al., 2016). Horizontal transfer of genes are responsible for acquiring virulence factors, antimicrobial genes, etc. (Hammerl et al., 2016). *Brucella* phage BiPBO1 is a temperate phage belonging to Siphoviridae, which was reported to infect various *Brucella* spp., including *B. melitensis* (Hammerl et al., 2016). *Brucella* phage BiPBO1 was predicted to be in an active state in all the 19 strains, but the role of this phage in these strains needs to be confirmed experimentally. Hammerl et al. (2016) reported that the attachment site for *Brucella* phage BiPBO1 is present in several *Brucella* spp., which supports the presence of this phage in all the 19 *B. melitensis* genome. Though phages of other bacteria are predicted in *B. melitensis*, their role is not clear, hence, requiring further experimental studies.

**FIGURE 3:**
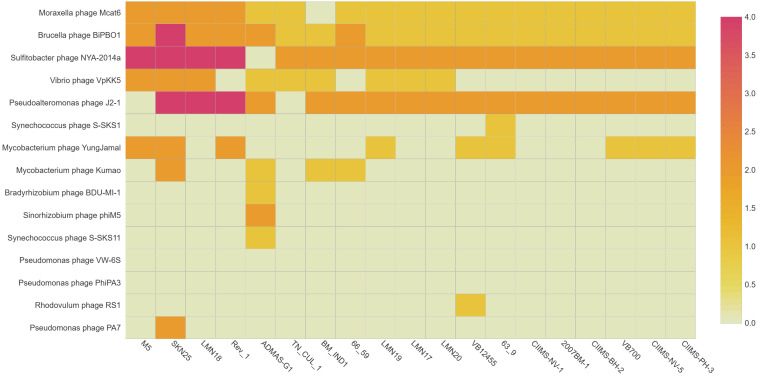
Prophages identified among the 19-genome analyzed.

Virulence factor genes are involved in synthesis of lipopolysaccharide (LPS), intracellular survival, iron uptake, secretion system, and motility/flagellar genes for *Brucella* (Rajendhran, 2021). A total of 43 virulence factor genes were predicted in all strains except for strain 2007BM-1 (*ricA* and *wbkA* are absent). RicA protein is involved in intracellular trafficking of *Brucella* spp. since interaction between RicA and Rab2 of host was found to be essential for translocation of the pathogen. It is reported that *ricA* mutation does not have a major impact on the replication of *Brucella* spp. inside the *Brucella* containing vacuoles, but there was alteration in the intracellular trafficking (de Barsy et al., 2011). Although there was absence of *ricA* gene, the presence of other genes like *manA* and *perA* in *B. melitensis* strain 2007BM-1 might be involved in the intracellular trafficking of the organism (Singh et al., 2018). *wbkA* is a glycosyltransferase/mannosyltransferase that is part of the synthesis of *O*-polysaccharide of LPS. The absence of this gene may lead to rough mutants, but the presence of other genes like *wboA* may compensate for the loss of *wbkA*, maintaining a smooth phenotype (Mancilla et al., 2012). Among all these isolates, ADMAS-G1 had higher variation in most of the virulence genes. This could be attributed to the unique clustering of the ADMAS-G1 strain with the American lineage compared with other strains from India (Figure 1). Other strains showing variability were SKN25, VB700, and VB12455 (Figure 3).

## Core and Accessory Gene Compositions

Panaroo pangenome analysis revealed 2,988 core genes and 188 accessory genes among a total of 3,176 gene pan-genome. This indicated that the *B. melitensis* has a highly conserved core genome structure. Multidimensional scaling indicated four clades among the 19 strains analyzed. Majority of the Indian strains (*n* = 13) including the vaccine strain M5 clustered in a single clade. Among the three other clades, two clades were represented by one isolate each, namely, ADMAS-G1 and Rev.1 from small ruminants. The fourth clade is represented by all four strains recovered from the state of Punjab (LNM17, LMN18, LNM19, and LMN20). Fripan dendogram and accessory genome plot revealed ADMAS-G1 forming an outgroup and harbor most unique genes compared with other strains.

**FIGURE 4:**
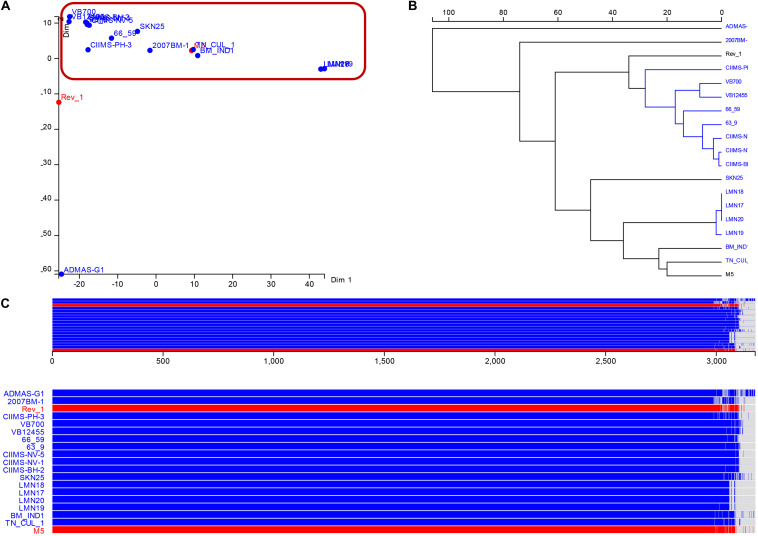
Results of pangenome analysis **(A)**. Multidimensional scaling **(B)**. Pangenome phylogeny **(C)**. Schematic representation of core and accessory genes among the various genomes analyzed. Indian strains are depicted in blue color and vaccine strains in red color font.

**FIGURE 5:**
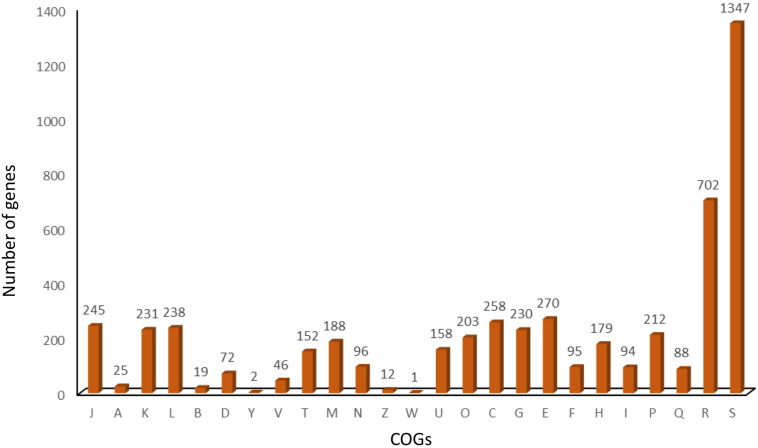
COG-based functional categories of core genome of the analyzed *Brucella melitensis*.

The multidimensional scaling and schematic representation of pan-genome are depicted in Figure 4. Pangenome analysis also indicated that certain genes were absent from strains of Punjab origin. Genes like *hyaD*, *rfbC*, *dapH_2*, *ompA_2*, *hldD_2*, *gmd_2*, *gumH*, and *wzxC* were found to be absent in Punjab strains (Supplementary Table 3). Genes like *hyaD*, which is a hyaluronan synthase, *rfbC*, a dTDP-4-dehydrorhamnose 3,5-epimerase, and *gmd*, a GDP-mannose 4,6-dehydratase were not found in *Brucella abortus* strains (*n* = 3), while they were present in *B. melitensis* 16M and 23457 as reported earlier in a study (Wattam et al., 2009). These genes were reported in a 23-kb segment region of *Brucella* spp. (Wattam et al., 2009). COG analysis of core genome indicates that most of the genes were of general function genes (*n* = 702) followed by amino acid transport and metabolism (*n* = 270), energy production and conversion (*n* = 258), translation, ribosomal structure, and biogenesis (*n* = 245). A total of 46 genes responsible for defense mechanism were also predicted in the core genome. COG-based functional categories of core genome of the analyzed *B. melitensis* are depicted in Figure 5.

Accessory genome encodes mostly genes of phages, mobile genetic elements, plasmids, etc., which are essential for colonizing the new niche (Bobay et al., 2013). COG annotation and classification of accessory genes revealed that majority of the genes are transposase and integrase, which were involved in recombination and DNA repair (L) COG category. Cell wall/membrane synthesis (M) genes like glycosyl transferase and acetyltransferase were predicted in accessory genomes. Several phage head and tail protease proteins, AMR genes, and metal resistance-associated genes with many regulatory proteins were predicted in the accessory genome (Supplementary Table 4). Phage proteins, insertional sequences predicted in accessory genome, and prophages predicted in the individual genome may have a role in the diversity of *B. melitensis*. Suárez-Esquivel et al. (2020) reported that insertional sequences in *Brucella* spp. may have a role in diversity within the species.

### Multilocus Sequence Typing Genotypes and Core Genome Phylogeny

**FIGURE 6:**
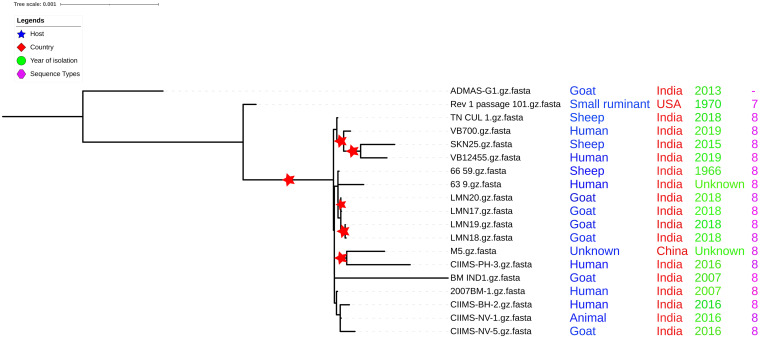
Core genome phylogeny with multilocus sequence typing (MLST) profiling. All Indian strains belong to ST8 except ADMAS-G1. Two subclusters were found in core genome phylogeny.

Multilocus sequence typing revealed that all the 17 Indian strains analyzed in the present study belonged to ST8 with the exception of strain ADMAS-G1 for which the complete allelic profile was not revealed. It is a four-locus variant (*aroA*, *trpE*, *cobQ*, and *omp25*) of ST8 (Indian strains) and double locus variant (*trpE* and *cobQ*) of ST7 (16M, Rev.1 strain) (Figure 6). Among the vaccine strains, the Chinese vaccine candidate M5 shared the same ST with that of Indian strains, whereas Rev.1 was recognized as ST7 (Supplementary Table 5). MLST-based analysis, hence, indicated higher conservation of Indian strains from different geographical lineages and hosts. Indian strains share the same ST with M5 indicating closely related evolutionary origins, whereas they are recognized as triple locus variants (TLVs) with vaccine strain Rev.1 and 16M (ST7) indicating distantly related evolutionary origins. Based on whole genome analysis of *B. melitensis*, ST7 was reported only in American lineages, while ST8 was reported from East Mediterranean lineages (Janowicz et al., 2020). As per the genome data in PubMLST database,^9^ the worldwide highest number of *B. melitensis* isolates (106 out of 234) were typed as ST8, and none of the isolates from Asia were typed as ST7. Isolates of East Mediterranean lineages including Indian and Chinese strains were of ST8, indicating that similar strains are distributed in the Asian continent (Sun et al., 2017). PubMLST also shows that out of the 11 *B. melitensis* strains from India, 7 isolates were typed as ST8, 2 were typed as ST12 (double locus variant of ST8), and 1 each belongs to ST11 and ST41, which both are single locus variant of ST8. Although the strain ADMAS-G1 was typed as ST7 by earlier workers, the epidemiological source for this isolate is not clear (Shome et al., 2016).

Furthermore, the genetic relatedness among Indian strains was inferred based on core genome phylogeny. For inferring the phylogeny, all the sites undergoing recombination were removed as recommended (Posada and Crandall, 2002) following recombinant site prediction with Gubbins (Croucher et al., 2015). No or a few recombinations were predicted in other Indian strains and Rev.1 (Supplementary Table 6). This indicates that recombination may not have a significant role in the evolution of *B. melitensis* Indian strains. It was also reported that recombination has less frequent influence than mutation in the evolution of *Brucella* spp. (Vishnu et al., 2015). Recent studies highlight the application of core genome phylogeny that involves all the genes shared by closely related strains for delineating genetic diversity (Mercier-Darty et al., 2020). Core genome-based phylogeny was created using ML approach highlighting bootstrap support values (Figure 6). The phylogeny indicated that the ADMAS-G1 and Rev.1 strains form outgroup cluster in comparison with other strains from India. Core genome phylogeny showed two major clusters with more than 70% bootstrap values, cluster I with all three Tamil Nadu strains and one Gujarat isolate, and another cluster (II) formed with the rest of the strains. Cluster II is further divided into two subgroups. Subgroup one clusters with all four Punjab strains, and subgroup two was shared by all four Maharashtra strains, one each from Karnataka and Andhra Pradesh strains. BMIND1 isolate from Andhra Pradesh showed high branch length evolution. Further analysis was carried out with SNP level to identify the genes involved in genetic variations among Indian strains.

### SNP Diversity and Genetic Relatedness of Indian Strains to Vaccine Strains

The *B. melitensis* genome is conserved since there is no other extrachromosomal material (plasmids), and there is limited report about the horizontal gene transfer, which may be due to its intracellular survival. So the other possible mechanism for the evolution of *B. melitensis* may be mutations so as to survive in a new geographical region and also to survive in a new host (Tan et al., 2015; Suárez-Esquivel et al., 2020). Hence, identifying the SNPs can enable to know the evolution of *B. melitensis* strains. Among several approaches, reference-free, alignment-free methods are suitable for many bacterial pathogens (Gardner et al., 2015). Also, pangenome SNP-based analysis can be used as a tool for molecular surveillance of the *B. melitensis* strains circulating in a geographical location. The analysis indicated a total of 3,400 SNPs, of which 1,026 were synonymous (S) and 1,695 were non-synonymous (NS). The NS/S ratio was 1.65 indicating diversifying population/positive selection and suggests that positive selection has role in evolution of *B. melitensis*. An earlier report also documents positive selection in *Brucella* genome reporting the role of positive selection in evolution (Vishnu et al., 2015). Excluding the SNPs unique to Rev.1, ADMAS-G1, and 16M-cluster outgroup-1987 recognized as an outliner, there were 453 SNPs identified in the Indian strains (16) of which 224 and 138 SNPs were non-synonymous and synonymous, respectively.

**FIGURE 7:**
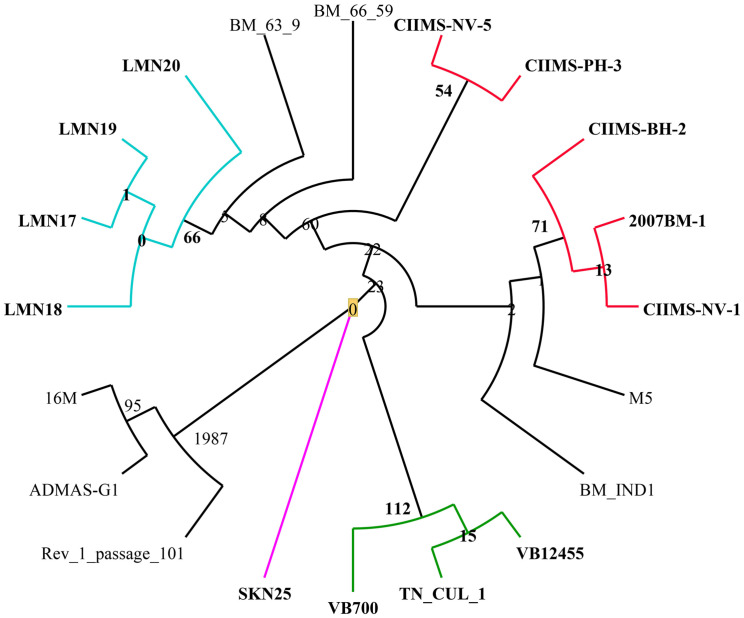
SNP-based phylogeny. Most of the Indian isolates except ADMAS-G1 clustered together. Blue, red, green, and pink color branches indicate isolates from Punjab, Maharashtra, Tamil Nadu, and Gujarat, respectively. Numbers in each node represent the node SNPs.

SNP-based phylogeny indicated geographical-wise grouping of isolates in India. The SNPs defining the clusters (node SNPs) and strain-specific SNPs (allele specific SNPs) are shown in Figure 7. Among them, the highest number of node SNPs (112) were observed for Tamil Nadu strains (correlated Cluster 1 in core genome phylogeny), which involved 24 synonymous SNPs and 44 non-synonymous SNPs. Punjab isolates were sharing 53 SNPs including 22 synonymous and 31 non-synonymous SNPs. Two isolates from Maharashtra (CIIMS-PH-3 and CIIMS-NV-5) shared 54 SNPs (21 synonymous and 23 non-synonymous) and another two isolates of Maharashtra (CIIMS-NV-1 and CIIMS-BH-2) along with one Karnataka isolate (2007BM-1) grouped together with 71 SNPs (21 synonymous and 33 non-synonymous). The Tamil Nadu cluster had non-synonymous SNPs in several peptidases like U32, M16 inactive domain protein, clp protease family protein, and M23 family protein. Non-synonymous SNPs were predicted in *motB* gene that is involved in chemotaxis and also in ABC transporter family proteins. *B. melitensis* is non-motile, but genes responsible for motility are reported, and *Brucella* mutants lacking *motB*, *fliF*, *flhA*, *fliC*, *flgE*, and *flgI* were found to be attenuated (Lestrate et al., 2003). M5 strain was also reported to have SNP in *motB* gene (Jiang et al., 2013). Punjab cluster had non-synonymous SNP in ribosomal protein L7/L12. This protein is essential for perfect translation of proteins, and it is also a major immunogen conferring protective immunity in murine models (Oliveira et al., 1996). In general, the genes involved in biosynthesis of amino acids, metabolism like cobalamin biosynthesis protein CobW, methionine synthase, arginase family protein, tryptophan synthase, alpha subunit, and glutamate-cysteine ligase, had non-synonymous SNPs in CIIMS-PH-3 and CIIMS-NV-5 cluster. CIIMS-BH-2 and CIIMS-NV-1 had non-synonymous mutation in lysE type translocator family protein. It was reported that *B. melitensis* Rev.1 had a mutation in lysE type translocator family protein when compared with 16M strain (Salmon-Divon et al., 2018).

The vaccine strains (M5-138, Rev.1-109) showed a high number of strain-specific SNPs compared with the other Indian strains except SKN25 (138) and BMIND1 (119) (Figure 7). Most of the Indian isolates except ADMAS-G1 are more closely related to vaccine strain M5. These strains that must have the same origin and trade between Asian countries might be a reason for the genetic relatedness of Indian strains with Chinese strains including M5 (Tan et al., 2015).

In the year 1957, *B. melitensis* Rev.1 vaccine strain was developed by serial passage of virulent strain 6056 on media containing streptomycin. Later in the year 1970, the Rev.1 strain underwent further passing, and passage 101 was used as stock culture. Apart from disadvantages like inducing abortion, it was reported that Rev.1 strain can have varying phenotypic and immunological properties impeding its use as an effective vaccine (Bosseray, 1991). Similarly, another vaccine strain M5 used in China was passaged 90 times in chicken embryo fibroblast (Research Group of Brucellosis, 1991). Both these vaccine strains do not have the potential to differentiate between vaccinated and infected animals, and both vaccines were reported to cause abortion (Jiménez de Bagués et al., 1989; Ficht et al., 2009). Since LPS is the major virulence factor of *Brucella* spp., various mutants lacking genes involved in LPS synthesis has been developed and evaluated for its potential as a safe vaccine. Rev. 1 mutants like *wbkF* mutant, *per* mutant, and *wa* mutant were developed (Barrio et al., 2009). Similarly, *manB* and *wboA* gene mutants were developed from M5 strain and were found as an effective vaccine candidate (Li et al., 2015; Zhang et al., 2017). Indian strain 2007BM-1 lack *ricA* and *wbkA* genes, which are essential virulence factors responsible for intracellular trafficking and LPS synthesis, respectively.

## Conclusion

Genome analysis of *B. melitensis* TN_CUL_1 predicted several virulence genes, prophage regions, etc. Comparative genomics of Indian strains showed that all strains shared same sequence type except ADMAS-G1. Genome composition analysis of virulence genes and prophages showed a conserved nature among isolates indicative of their stable role in the evolution of *B. melitensis.* Recombination events were less and, hence, indicated that mutational events were predominant in strain diversification. Comparison with strains based on core genome phylogeny showed better strain discrimination compared with MLST sequence types. The core genome and SNP-based phylogeny indicated two to three well-defined groups with strong bootstrap support. The TN_CUL_1 strain-involved cluster (three strains) was forming a separate outgroup indicative of possible different evolutionary lineages within India. The core genome phylogeny and SNP analysis indicated that Indian strains were related to M5 compared with Rev.1. Hence, any Indian field strains from small ruminants may be targeted for developing a novel vaccine candidate for small ruminant brucellosis in India.

## Data Availability Statement

The data presented in the study are deposited in the NCBI SRA repository with the accession number NZ_JACNEQ000000000.1 and assembly number GCA_014270005.1.

## Author Contributions

KK, SA, and PT performed the sequence data analysis and comparative genomics, and drafted the manuscript. MA, TS, and RS carried out the identification of virulence genes and prophages. GD critically evaluated and edited the manuscript. All authors contributed to the article and approved the submitted version.

## Conflict of Interest

The authors declare that the research was conducted in the absence of any commercial or financial relationships that could be construed as a potential conflict of interest.

## Publisher’s Note

All claims expressed in this article are solely those of the authors and do not necessarily represent those of their affiliated organizations, or those of the publisher, the editors and the reviewers. Any product that may be evaluated in this article, or claim that may be made by its manufacturer, is not guaranteed or endorsed by the publisher.

## Footnotes

1
https://galaxyproject.org

2
https://usegalaxy.org

3
http://www.bioinformatics.babraham.ac.uk/projects/fastqc/

4
https://realphy.unibas.ch/realphy/

5
https://app.displayr.com/

6
https://github.com/drpowell/FriPan

7
http://eggnog-mapper.embl.de

8
https://pubmlst.org/organisms/brucella-spp

9
https://pubmlst.org/brucella/

## Supplementary Material

Supplementary Figure 1 Circular plot of chromosome I **(A)** and II **(B)** with CDS of TN_CUL_1 strain.

Supplementary Table 1 The available genome assembly data for *Brucella melitensis* (355) as on January 26th, 2021 from NCBI were retrieved.

Supplementary Table 2 Metadata of Indian strains (*n* = 17) and vaccines strains Rev.1 and M5.

Supplementary Table 3 Gene presence and absence as per pangenome analysis.

Supplementary Table 4 COG annotation and classification of accessory genes in pangenome analysis.

Supplementary Table 5 MLST profiling of the genome analyzed.

Supplementary Table 6 Table containing information regarding gubbins recombination prediction.

## References

[B1] Afgan E. Baker D. Batut B. van den Beek M. Bouvier D. Cech M. (2018). The Galaxy platform for accessible, reproducible and collaborative biomedical analyses: 2018 update. *Nucleic Acids Res.* 46 W537–W544. 29790989 10.1093/nar/gky379 PMC6030816

[B2] Alton G. G. (1990). “Brucella melitensis,” in *“Animal brucellosis*, eds Nielsen K. Duncan J. R. (Boston: CRC Press), 383–409.

[B3] Assefa S. Keane T. M. Otto T. D. Newbold C. Berriman M. (2009). ABACAS: algorithm-based automatic contiguation of assembled sequences. *Bioinformatics* 25 1968–1969. 10.1093/bioinformatics/btp347 19497936 PMC2712343

[B4] Azam S. Rao S. B. Jakka P. NarasimhaRao V. Bhargavi B. Gupta V. K. (2016). Genetic characterization and comparative genome analysis of *Brucella melitensis* isolates from India. *Int. J. Genomics* 2016:3034756. 10.1155/2016/3034756 27525259 PMC4976149

[B5] Aziz R. K. Bartels D. Best A. A. DeJongh M. Disz T. Edwards R. A. (2008). The RAST Server: rapid annotations using subsystems technology. *BMC Genomics* 9:75. 10.1186/1471-2164-9-75 18261238 PMC2265698

[B6] Bardenstein S. Mandelboim M. Ficht T. A. Baum M. Banai M. (2002). Identification of the *Brucella melitensis* vaccine strain Rev.1 in animals and humans in Israel by PCR analysis of the PstI site polymorphism of its omp2 gene. *J. Clin. Microbiol.* 40 1475–1480. 10.1128/jcm.40.2.1475-1480.2002 11923376 PMC140367

[B7] Barrio M. B. Grilló M. J. Muñoz P. M. Jacques I. González D. de Miguel M. J. (2009). Rough mutants defective in core and O-polysaccharide synthesis and export induce antibodies reacting in an indirect ELISA with smooth lipopolysaccharide and are less effective than Rev 1 vaccine against *Brucella melitensis* infection of sheep. *Vaccine* 27 1741–1749. 10.1016/j.vaccine.2009.01.025 19186196

[B8] Blasco J. M. Molina-Flores B. (2011). Control and eradication of *Brucella melitensis* infection in sheep and goats. *Vet. Clin. North Am. Food Anim. Pract.* 27 95–104. 10.1016/j.cvfa.2010.10.003 21215893

[B9] Bobay L. M. Rocha E. P. Touchon M. (2013). The adaptation of temperate bacteriophages to their host genomes. *Mol. Biol. Evol.* 30 737–751. 10.1093/molbev/mss279 23243039 PMC3603311

[B10] Bosseray N. (1991). *Brucella melitensis* Rev. 1 living attenuated vaccine: stability of markers, residual virulence and immunogenicity in mice. *Biologicals* 19 355–363. 10.1016/s1045-1056(05)80025-9 1797046

[B11] Bricker B. J. Halling S. M. (1994). Differentiation of *Brucella abortus* bv. 1, 2, and 4, *Brucella melitensis*, *Brucella ovis*, and *Brucella suis* bv. 1 by PCR. *J. Clin. Microbiol.* 32 2660–2666. 10.1128/JCM.32.11.2660-2666.1994 7852552 PMC264138

[B12] Croucher N. J. Page A. J. Connor T. R. Delaney A. J. Keane J. A. Bentley S. D. (2015). Rapid phylogenetic analysis of large samples of recombinant bacterial whole genome sequences using Gubbins. *Nucleic Acids Res.* 43:e15. 10.1093/nar/gku1196 25414349 PMC4330336

[B13] de Barsy M. Jamet A. Filopon D. Nicolas C. Laloux G. Rual J. F. (2011). Identification of a *Brucella* spp. secreted effector specifically interacting with human small GTPase Rab2. *Cell Microbiol.* 13 1044–1058. 10.1111/j.1462-5822.2011.01601.x 21501366

[B14] Doganay G. D. Doganay M. (2013). Brucella as a potential agent of bioterrorism. *Recent Pat. Antiinfect. Drug Discov*. 8 27–33. 10.2174/1574891x11308010006 22934672

[B15] Ficht T. A. Kahl-McDonagh M. M. Arenas-Gamboa A. M. Rice-Ficht A. C. (2009). Brucellosis: the case for live, attenuated vaccines. *Vaccine* 27 D40–D43. 10.1016/j.vaccine.2009.08.058 19837284 PMC2780424

[B16] Gardner S. N. Slezak T. Hall B. G. (2015). kSNP3.0: SNP detection and phylogenetic analysis of genomes without genome alignment or reference genome. *Bioinformatics* 31 2877–2878. 10.1093/bioinformatics/btv271 25913206

[B17] Hammerl J. A. Göllner C. Al Dahouk S. Nöckler K. Reetz J. Hertwig S. (2016). Analysis of the First Temperate Broad Host Range Brucellaphage (BiPBO1) Isolated from *B. inopinata*. *Front. Microbiol.* 7:24. 10.3389/fmicb.2016.00024 26858702 PMC4729917

[B18] Janowicz A. De Massis F. Zilli K. Ancora M. Tittarelli M. Sacchini F. (2020). Evolutionary history and current distribution of the West Mediterranean lineage of *Brucella melitensis* in Italy. *Microb. Genom.* 6:mgen000446. 10.1099/mgen.0.000446 33030422 PMC7725330

[B19] Jiang H. Du P. Zhang W. Wang H. Zhao H. Piao D. (2013). Comparative genomic analysis of *Brucella melitensis* vaccine strain M5 provides insights into virulence attenuation. *PLoS One* 8:e70852. 10.1371/journal.pone.0070852 23967122 PMC3743847

[B20] Jiménez de Bagués M. P. Marin C. M. Barberán M. Blasco J. M. (1989). Responses of ewes to *B. melitensis* Rev1 vaccine administered by subcutaneous or conjunctival routes at different stages of pregnancy. *Ann. Rech. Vet.* 20 205–213. 2751232

[B21] Kaden R. Ågren J. Båverud V. Hallgren G. Ferrari S. Börjesson J. (2014). Brucellosis outbreak in a Swedish kennel in 2013: determination of genetic markers for source tracing. *Vet. Microbiol.* 174 523–530. 10.1016/j.vetmic.2014.10.015 25465667

[B22] Khan A. U. Melzer F. Sayour A. E. Shell W. S. Linde J. Abdel-Glil M. (2021). Whole-Genome Sequencing for Tracing the Genetic Diversity of *Brucella abortus* and *Brucella melitensis* Isolated from Livestock in Egypt. *Pathogens* 10:759. 10.3390/pathogens10060759 34208761 PMC8235727

[B23] Kishnani P. M. Tiwari A. A. Mangalgi S. S. Barbuddhe S. B. Daginawala H. F. Singh L. R. (2018). Whole-Genome Sequence of *Brucella melitensis* CIIMS-BH-2, a Biovar 2 Strain Isolated from Human Blood. *Genome Announc*. 6 e00079–18. 10.1128/genomeA.00079-18 29519823 PMC5843732

[B24] Lestrate P. Dricot A. Delrue R. M. Lambert C. Martinelli V. De Bolle X. (2003). Attenuated signature- tagged mutagenesis mutants of *Brucella melitensis* identified during the acute phase of infection in mice. *Infect. Immun.* 71 7053–7060. 10.1128/iai.71.12.7053-7060.2003 14638795 PMC308902

[B25] Letunic I. Bork P. (2007). Interactive Tree Of Life (iTOL): an online tool for phylogenetic tree display and annotation. *Bioinformatics* 23 127–128. 10.1093/bioinformatics/btl529 17050570

[B26] Li Z. Q. Shi J. X. Fu W. D. Zhang Y. Zhang J. Wang Z. (2015). A *Brucella melitensis* M5-90 wboA deletion strain is attenuated and enhances vaccine efficacy. *Mol. Immunol.* 66 276–283. 10.1016/j.molimm.2015.04.004 25899866

[B27] Liu B. Zheng D. Jin Q. Chen L. Yang J. (2019). VFDB 2019: a comparative pathogenomic platform with an interactive web interface. *Nucleic Acids Res.* 47 D687–D692. 30395255 10.1093/nar/gky1080 PMC6324032

[B28] Mancilla M. Marín C. M. Blasco J. M. Zárraga A. M. López-Goñi I. Moriyón I. (2012). Spontaneous excision of the O-polysaccharide wbkA glycosyltranferase gene is a cause of dissociation of smooth to rough *Brucella* colonies. *J. Bacteriol.* 194 1860–1867. 10.1128/JB.06561-11 22328663 PMC3318470

[B29] Mantur B. G. Amarnath S. K. (2008). Brucellosis in India – a review. *J. Biosci*. 33 539–547. 10.1007/s12038-008-0072-1 19208979

[B30] Martins Rda C. Irache J. M. Gamazo C. (2012). Acellular vaccines for ovine brucellosis: a safer alternative against a worldwide disease. *Expert Rev. Vaccines* 11 87–95. 10.1586/erv.11.172 22149711

[B31] Mercier-Darty M. Royer G. Lamy B. Charron C. Lemenand O. Gomart C. (2020). Comparative whole-genome phylogeny of animal, environmental, and human strains confirms the genogroup organization and diversity of the *Stenotrophomonas maltophilia* complex. *Appl. Environ. Microbiol.* 86 e2919–19. 10.1128/AEM.02919-19 32198168 PMC7205487

[B32] Office International des Epizooties (OIE). (2018). *Brucellosis (Brucella abortus, B. melitensis and B. suis) (Infection with B. abortus, B. melitensis and B. suis).* Paris: OIE Terrestrial Manual.

[B33] Oliveira S. C. Harms J. S. Banai M. Splitter G. A. (1996). Recombinant Brucella abortus proteins that induce proliferation and gamma-interferon secretion by CD4+ T cells from Brucella-vaccinated mice and delayed-type hypersensitivity in sensitized guinea pigs. *Cell. Immunol.* 172 262–268. 10.1006/cimm.1996.0241 8964089

[B34] Pisarenko S. V. Kovalev D. A. Volynkina A. S. Ponomarenko D. G. Rusanova D. V. Zharinova N. V. (2018). Global evolution and phylogeography of *Brucella melitensis* strains. *BMC Genomics* 19:353. 10.1186/s12864-018-4762-2 29747573 PMC5946514

[B35] Posada D. Crandall K. A. (2002). The effect of recombination on the accuracy of phylogeny estimation. *J. Mol. Evol.* 54 396–402. 10.1007/s00239-001-0034-9 11847565

[B36] Rajendhran J. (2021). Genomic insights into Brucella. *Infect. Genet. Evol.* 87:104635. 10.1016/j.meegid.2020.104635 33189905

[B37] Research Group of Brucellosis (Harbin Veterinary Research Institute). (1991). Study on the Brucella melitensis strain M5-90 vaccine. *Chin. J. Control Endemic Dis.* 6 65–68.

[B38] Salmon-Divon M. Yeheskel A. Kornspan D. (2018). Genomic analysis of the original Elberg *Brucella melitensis* Rev.1 vaccine strain reveals insights into virulence attenuation. *Virulence* 9 1436–1448. 10.1080/21505594.2018.1511677 30139304 PMC6141144

[B39] Schaeffer J. Revilla-Fernández S. Hofer E. Posch R. Stoeger A. Leth C. (2021). Tracking the Origin of Austrian Human Brucellosis Cases Using Whole Genome Sequencing. *Front. Med*. 8:635547. 10.3389/fmed.2021.635547 33718408 PMC7943447

[B40] Seemann T. (2014). Prokka: rapid prokaryotic genome annotation. *Bioinformatics* 30 2068–2069. 10.1093/bioinformatics/btu153 24642063

[B41] Shome R. Gupta V. K. Rao K. N. Shome B. R. Nagalingam M. Rahman H. (2014). Detection of *Brucella melitensis* Rev–1 vaccinal antibodies in sheep in India. *Adv. Anim. Vet. Sci.* 2 19–22. 10.14737/journal.aavs/2014/2.3s.19.22

[B42] Shome R. Kalleshamurthy T. Rathore Y. Ramanjinappa K. D. Skariah S. Nagaraj C. (2020). Spatial sero-prevalence of brucellosis in small ruminants of India: nationwide cross-sectional study for the year 2017-2018. *Transbound. Emerg. Dis.* 68 2199–2208. 10.1111/tbed.13871 33021085

[B43] Shome R. Krithiga N. Shankaranarayana P. B. Jegadesan S. Udayakumar S. V. Shome B. R. (2016). Genotyping of Indian antigenic, vaccine, and field *Brucella* spp. using multilocus sequence typing. *J. Infect. Dev. Ctries.* 10 237–244. 10.3855/jidc.6617 27031455

[B44] Simpson G. Thompson P. N. Saegerman C. Marcotty T. Letesson J. J. de Bolle X. (2021). Brucellosis in wildlife in Africa: a systematic review and meta-analysis. *Sci. Rep.* 11:5960. 10.1038/s41598-021-85441-w 33727580 PMC7966391

[B45] Singh D. K. Kumar B. Shrinet G. Singh R. P. Das A. Mantur B. G. (2018). Draft genome sequence of field isolate Brucella melitensis strain 2007BM/1 from India. *J. Glob. Antimicrob. Resist.* 13 152–153. 10.1016/j.jgar.2018.04.008 29684575

[B46] Song W. Sun H. X. Zhang C. Cheng L. Peng Y. Deng Z. (2019). Prophage Hunter: an integrative hunting tool for active prophages. *Nucleic Acids Res.* 47 W74–W80. 31114893 10.1093/nar/gkz380 PMC6602508

[B47] Suárez-Esquivel M. Chaves-Olarte E. Moreno E. Guzmán-Verri C. (2020). *Brucella* Genomics: macro and Micro Evolution. *Int. J. Mol. Sci.* 21:7749. 10.3390/ijms21207749 33092044 PMC7589603

[B48] Sun M. Jing Z. Di D. Yan H. Zhang Z. Xu Q. (2017). Multiple Locus Variable-Number Tandem-Repeat and Single-Nucleotide Polymorphism-Based *Brucella* Typing Reveals Multiple Lineages in *Brucella melitensis* Currently Endemic in China. *Front. Vet. Sci.* 4:215. 10.3389/fvets.2017.00215 29312964 PMC5735110

[B49] Tan K. K. Tan Y. C. Chang L. Y. Lee K. W. Norel S. S. Yee W. Y. (2015). Full genome SNP-based phylogenetic analysis reveals the origin and global spread of *Brucella melitensis*. *BMC Genomics* 16:93. 10.1186/s12864-015-1294-x 25888205 PMC4409723

[B50] Tatusova T. DiCuccio M. Badretdin A. Chetvernin V. Nawrocki E. P. Zaslavsky L. (2016). NCBI prokaryotic genome annotation pipeline. *Nucleic Acids Res.* 44 6614–6624. 10.1093/nar/gkw569 27342282 PMC5001611

[B51] Tonkin-Hill G. MacAlasdair N. Ruis C. Weimann A. Horesh G. Lees J. A. (2020). Producing polished prokaryotic pangenomes with the Panaroo pipeline. *Genome Biol.* 21:180. 10.1186/s13059-020-02090-4 32698896 PMC7376924

[B52] Tormo M. Á Ferrer M. D. Maiques E. Úbeda C. Selva L. Lasa Í (2008). *Staphylococcus aureus* pathogenicity island DNA is packaged in particles composed of phage proteins. *J. Bacteriol.* 190 2434–2440. 18223072 10.1128/JB.01349-07 PMC2293202

[B53] Trifinopoulos J. Nguyen L. T. von Haeseler A. Minh B. Q. (2016). W-IQ-TREE: a fast online phylogenetic tool for maximum likelihood analysis. *Nucleic Acids Res.* 44 W232–W235. 27084950 10.1093/nar/gkw256 PMC4987875

[B54] Tsang A. K. L. Lee H. H. Yiu S. M. Lau S. K. P. Woo P. C. Y. (2017). Failure of phylogeny inferred from multilocus sequence typing to represent bacterial phylogeny. *Sci. Rep.* 7:4536. 10.1038/s41598-017-04707-4 28674428 PMC5495804

[B55] Vishnu U. S. Sankarasubramanian J. Sridhar J. Gunasekaran P. Rajendhran J. (2015). Identification of Recombination and Positively Selected Genes in Brucella. *Indian J. Microbiol*. 55 384–391. 10.1007/s12088-015-0545-5 26543263 PMC4627946

[B56] Wang X. Wood T. K. (2016). Cryptic prophages as targets for drug development. *Drug Resist. Updat.* 27 30–38. 10.1016/j.drup.2016.06.001 27449596

[B57] Wattam A. R. Williams K. P. Snyder E. E. Almeida N. F. Jr. Shukla M. Dickerman A. W. (2009). Analysis of ten *Brucella* genomes reveals evidence for horizontal gene transfer despite a preferred intracellular lifestyle. *J. Bacteriol.* 191 3569–3579. 19346311 10.1128/JB.01767-08 PMC2681906

[B58] Whatmore A. M. Perrett L. L. MacMillan A. P. (2007). Characterisation of the genetic diversity of Brucella by multilocus sequencing. *BMC Microbiol*. 7:34. 10.1186/1471-2180-7-34 17448232 PMC1877810

[B59] Wick R. R. Judd L. M. Gorrie C. L. Holt K. E. (2017). Unicycler: resolving bacterial genome assemblies from short and long sequencing reads. *PLoS Comput. Biol*. 13:e1005595. 10.1371/journal.pcbi.1005595 28594827 PMC5481147

[B60] Zhang J. Yin S. Yi D. Zhang H. Li Z. Guo F. (2017). The Brucella melitensis M5-90ΔmanB live vaccine candidate is safer than M5-90 and confers protection against wild-type challenge in BALB/c mice. *Microb. Pathog.* 112 148–155. 28916316 10.1016/j.micpath.2017.09.016

