# Supplementary figures and images for: Genome Sequencing and Comparative Genomics of Indian Isolates of Brucella melitensis
Source: Front Microbiol. 2021 Aug 20;12:698069. doi: 10.3389/fmicb.2021.698069 (PMC8417702; doi:10.3389/fmicb.2021.698069)

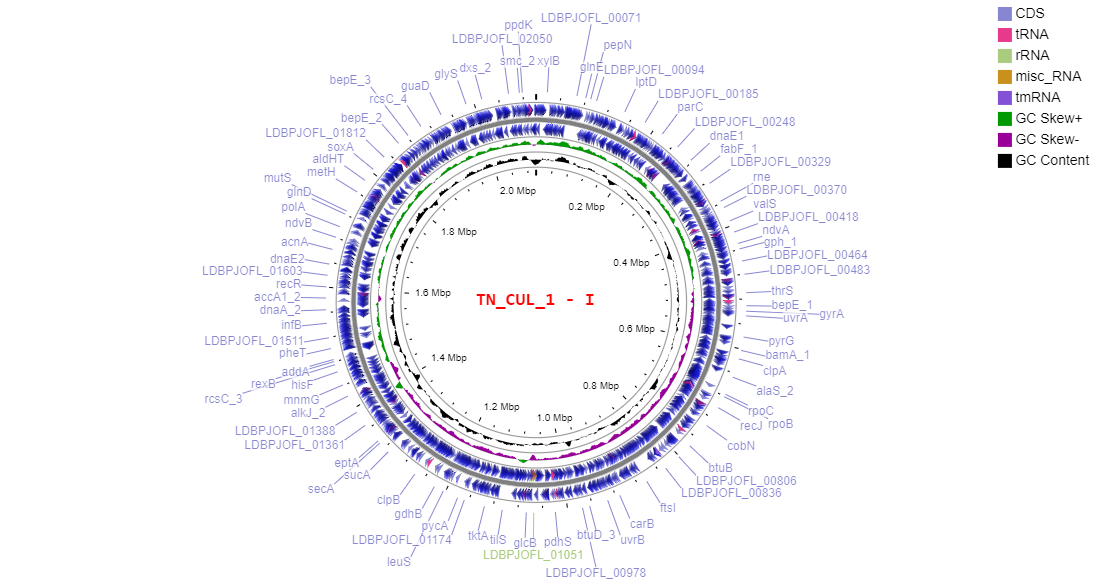

Supplement: Supplementary Figure 1 — Circular plot of chromosome I (A) and II (B) with CDS of TN_CUL_1 strain. [file Image_1.PNG]

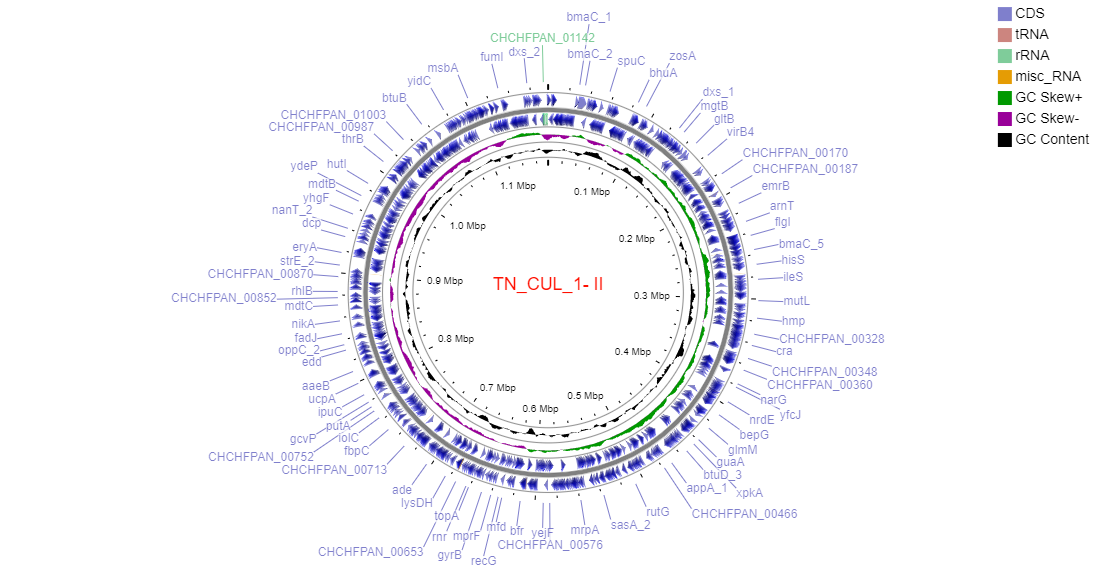

Supplement: Supplementary file 8 [file Image_2.PNG]
